# Sensory gating of an embryonic zebrafish interneuron during spontaneous motor behaviors

**DOI:** 10.3389/fncir.2014.00121

**Published:** 2014-09-30

**Authors:** Laura D. Knogler, Pierre Drapeau

**Affiliations:** Departments of Pathology and Cell Biology and Neuroscience, Centre hospitalier de l’Université de Montréal Research Centre and Le Groupe de Recherche sur le Système Nerveux Central, Université de MontréalMontréal, QC, Canada

**Keywords:** sensory interneurons, zebrafish, spinal cord, spontaneous behavior, glycine receptors, AMPA receptors, corollary discharge, reflex inhibition

## Abstract

In all but the simplest monosynaptic reflex arcs, sensory stimuli are encoded by sensory neurons that transmit a signal *via* sensory interneurons to downstream partners in order to elicit a response. In the embryonic zebrafish (*Danio rerio*), cutaneous Rohon-Beard (RB) sensory neurons fire in response to mechanical stimuli and excite downstream glutamatergic commissural primary ascending (CoPA) interneurons to produce a flexion response contralateral to the site of stimulus. In the absence of sensory stimuli, zebrafish spinal locomotor circuits are spontaneously active during development due to pacemaker activity resulting in repetitive coiling of the trunk. Self-generated movement must therefore be distinguishable from external stimuli in order to ensure the appropriate activation of touch reflexes. Here, we recorded from CoPAs during spontaneous and evoked fictive motor behaviors in order to examine how responses to self-movement are gated in sensory interneurons. During spontaneous coiling, CoPAs received glycinergic inputs coincident with contralateral flexions that shunted firing for the duration of the coiling event. Shunting inactivation of CoPAs was caused by a slowly deactivating chloride conductance that resulted in lowered membrane resistance and increased action potential threshold. During spontaneous burst swimming, which develops later, CoPAs received glycinergic inputs that arrived in phase with excitation to ipsilateral motoneurons and provided persistent shunting. During a touch stimulus, short latency glutamatergic inputs produced cationic currents through AMPA receptors that drove a single, large amplitude action potential in the CoPA before shunting inhibition began, providing a brief window for the activation of downstream neurons. We compared the properties of CoPAs to those of other spinal neurons and propose that glycinergic signaling onto CoPAs acts as a corollary discharge signal for reflex inhibition during movement.

## INTRODUCTION

Early embryonic circuits must be capable of responding to sensory stimuli in order to perform essential motor behaviors such as avoiding predation. Additionally, sensorimotor circuits must be organized in such a way that the animal’s own movement is distinguishable from movement in the environment in order to make computations regarding expected and novel sensory feedback. One way in which the nervous system compensates for self-movement is to send corollary discharge signals from motor-related pathways to modulate activation of sensory pathways (reviewed by Crapse and Sommer, 2008). Corollary discharges may target different parts of the sensory pathway and use diverse mechanisms to alter sensory function depending on the species and the modality being studied. The embryonic vertebrate spinal cord provides a simplified neural network within which to study the modulation of sensory pathways that ensure the appropriate activation of sensorimotor behaviors.

A simple disynaptic spinal reflex arc has been described for a contralateral flexion response in hatchling *xenopus* (Li et al., 2003). Following a touch to the skin, one or more Rohon-Beard (RB) sensory neurons strongly activate glutamatergic dorsolateral commissural (dlc) sensory interneurons which in turn excite premotor interneurons and motoneurons, leading to a contraction of the contralateral trunk away from the stimulus followed by escape swimming. Immediately following dlc interneuron excitation, glycinergic corollary discharges arrive in phase with excitation to ipsilateral motoneurons to provide sustained inhibition to the dlc interneuron during swimming (Sillar and Roberts, 1988; Soffe, 1993; Li et al., 2002, 2004). The relative abundance of AMPA and NMDA receptors in different neuronal classes within the circuit also contributes to the proper activation of this reflex pathway (Li et al., 2003).

In the zebrafish spinal cord, commissural primary ascending (CoPA) interneurons are one of the earliest born cells and extend long axons that span more than ten somites rostrally by 26 hours post-fertilization (hpf; Bernhardt et al., 1990), prior to hatching on day two. CoPAs are thought to be homologous to *xenopus* dlc interneurons due to their similarly large dorsal soma, ascending commissural axonal projections and glutamatergic identities (Bernhardt et al., 1990; Higashijima et al., 2004a). In addition, embryonic CoPAs are contacted by RB sensory neurons (Easley-Neal et al., 2013) and following a response to touch at 24 hpf receive short latency excitation followed by a long duration glycine-mediated conductance that is thought to inhibit activation (Pietri et al., 2009). Based on these results, zebrafish CoPAs are predicted to be sensory interneurons that carry the initial excitation to the contralateral spinal cord during evoked behaviors, as dlc interneurons do in *xenopus*. However, very little is known about the intrinsic and synaptic properties of CoPAs and their pattern of activation during spontaneous behaviors such as coiling and swimming.

We hypothesized that CoPAs must be selectively inhibited during early spontaneous coiling and later swimming behaviors in order to prevent the ongoing activation of the touch reflex during spontaneous movement. To test this idea, we targeted embryonic CoPA interneurons for electrophysiological recordings in order to investigate their intrinsic and synaptic properties in the context of spontaneous and touch-evoked behaviors. In particular, we looked for activation patterns and receptor properties of CoPAs that could contribute to their specialized role in gating somatosensory activation of a reflex pathway. We show that inhibitory corollary discharges onto CoPA interneurons combined with the presence of a rare glycine conductance with slow kinetics contribute to the inhibition of the somatosensory pathway during early spontaneous behaviors. These findings are compared to results from other vertebrates as well as invertebrates in order to determine general features of sensory gating in spinal circuits.

## MATERIALS AND METHODS

### ZEBRAFISH MAINTENANCE

Tübingen wildtype strains of adult zebrafish were maintained according to guidelines approved by the Animal Experimentation Ethics Committee, Université de Montréal. Staging of embryos (of as yet undetermined sex) was performed as previously described (Kimmel et al., 1995).

### ELECTROPHYSIOLOGY AND PHARMACOLOGY

Zebrafish embryos were dechorionated and anesthetized in 0.02% tricaine dissolved in Evans solution (134 mM NaCl, 2.9 mM KCl, 2.1 mM CaCl2, 1.2 mM MgCl2, 10 mM glucose, 10 mM HEPES, pH 7.8 with NaOH) and dissected according to previously described procedures (Drapeau et al., 1999). Briefly, spinal neurons in somites 5–15 were selected for recording based on their soma size and position as visualized by oblique illumination (Olympus BX61W1). To record spontaneous activity, 15 μM D-tubocurarine was added to the Evans solution to block neuromuscular transmission. To stimulate a touch response, a glass electrode connected to a picospritzer (Parker Hannifin, Fairfield, NJ, USA) squirted bath solution near the tip of the tail at the desired pressure and duration (5–10 psi, 4–20 ms).

Electrophysiological recordings were done in the presence of 1–10 μM strychnine to block glycine receptors, in 7–10 μM 6-cyano-7-nitroquinoxaline-2,3-dione (CNQX) to block AMPA receptors, or in 50 μM (2R)-amino-5-phosphonovaleric acid (APV) to block NMDA receptors. 0.5–1 μM tetrodotoxin (TTX) was used to block evoked activity when recording miniature post-synaptic currents. Patch-clamp electrodes for spinal neuron recordings (6–14 MΩ) were pulled from borosilicate glass and were filled with the following intracellular solution (in mM): 105 D-gluconic acid, 16 KCl, 2 MgCl2, 10 HEPES, and 10 EGTA, adjusted to pH 7.2, 290 mOsm (Drapeau et al., 1999). Sulforhodamine B (0.1%) was also included in the patch solution to label the cells and confirm their identity after a recording. Filled CoPAs had a triangular soma and distinctive dendrites that distinguished them from otherwise morphologically similar CoSAs. Recordings from cells whose identity was ambiguous were not included in subsequent analyses. 4 mM QX-314 was added to the intracellular solution in some experiments to block voltage-gated sodium channels. All drugs were obtained from Sigma (St. Louis, MS, USA).

Standard single and dual whole-cell recordings from 24 to 29 hpf larvae were obtained using an Axopatch 200B and a Molecular Devices CV 203BU headstage amplifier (Molecular Devices). Data were acquired at 40 kHz and low-pass filtered at 10 kHz. Cells were held near their resting potential at –65 mV under voltage clamp unless otherwise specified. A maximum of three neural recordings were obtained from each embryo. Electrophysiological analyses were performed oﬄine using Clampex 10.2 and Clampfit 10.2 software (Molecular Devices). The recordings were not analyzed if the resting membrane potential was more positive than –40 mV or if the input resistance was below 500 MΩ. Following each recording, a series of fluorescent images of the rhodamine-filled cell and its axonal projections as well as bright-field images were collected with a QImaging camera (model 1394, QImaging Corporation Canada) using Micro-Manager software (). Images were inverted and brightness/contrast was adjusted using Adobe Photoshop CS2 (Adobe Systems, Inc., San Jose, CA, USA).

### STATISTICAL ANALYSES

SPSS 21 (IBM) was used to assess data for statistical significance. All data sets were initially assessed for normality with the Shapiro–Wilk Test. For independent data sets with only two groups we used the Student’s *t*-test or Mann–Whitney *U* test, and for data from multiple groups we used a one-way ANOVA with Tukey’s *post hoc* test to look for differences between conditions (*p* < 0.05). In data obtained from the same group under different conditions, the Paired-samples *t*-test or repeated-measures variation of ANOVA was used. A Pearson statistic or Spearman rank rho was used to assess correlations for parametric or non-parametric data, respectively. Statistical significance is represented in the graphs as ∗∗∗ for *p* < 0.001, ∗∗ for *p* < 0.01, and ∗ for *p* < 0.05, and individual *p*-values and numbers of embryos per condition are provided in figure legends and the text. Error bars in bar graphs indicate the SE, and results are described as the mean ± SE. Excel was used to create all graphs except the box plots, which were created online using the BoxPlotR application (http://boxplot.tyerslab.com/). In these plots, box limits indicate the 25th and 75th percentiles as determined by R software, center lines show the medians, whiskers extend 1.5 times the interquartile range from the 25th and 75th percentiles and outliers are represented by open circles.

## RESULTS

### INTRINSIC PROPERTIES OF EMBRYONIC CoPA INTERNEURONS ARE SIMILAR TO OTHER SPINAL NEURONS

**FIGURE 1:**
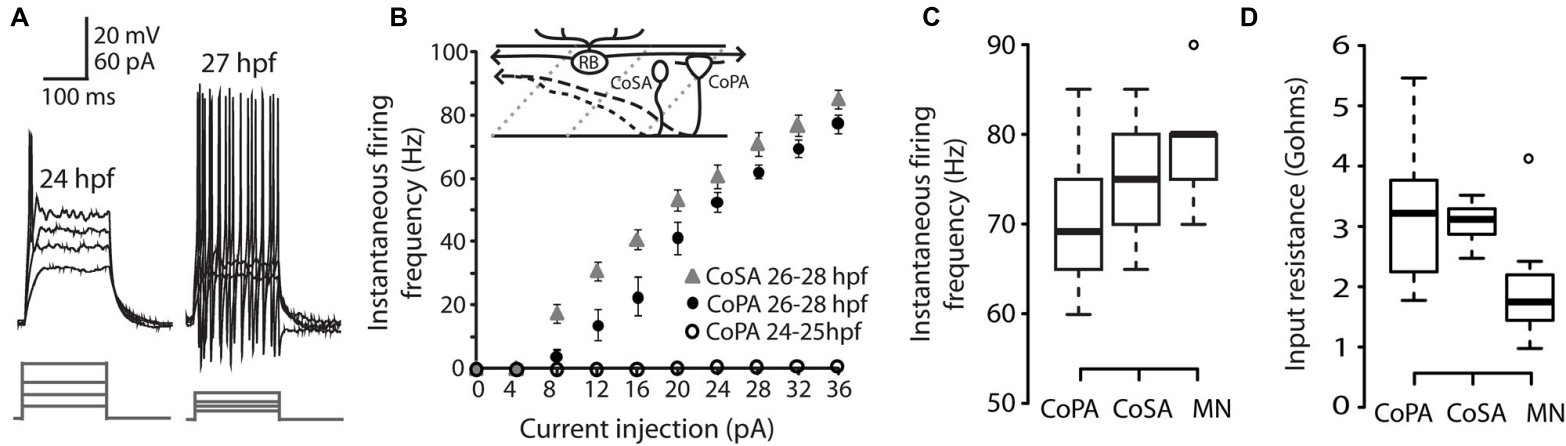
**Intrinsic properties of embryonic commissural primary ascending (CoPA) interneurons are similar to other spinal neurons. (A)** Representative current injections showing single vs. burst firing of action potentials in CoPAs at 24 and 27 hours post-fertilization (hpf), respectively. Upper traces, current-clamp recording, lower traces, current steps. Note the reduction in action potential threshold at 27 hpf. **(B)** Quantification of instantaneous firing frequency (Hz) vs. current injection (pA) for CoPA and CoSA neurons in 26–28 hpf embryos (*N* = 9, 9) and CoPA neurons in 24–25 hpf embryos (*N* = 5). Inset shows the general morphology of these spinal neurons and the sensory RB neuron that contacts CoPAs. In the drawing, rostral is to the left, dorsal is up, dotted gray lines indicate somite boundaries, and dashed black lines indicate commissural axonal projections. **(C)** Box plot showing the similarity of firing frequencies between CoPAs, CoSAs, and MNs in 26–28 hpf embryos in response to a 32 pA step of depolarizing current (*N* = 9, 5, 9; *p* > 0.05 for all pairwise comparisons). **(D)** Box plot showing the input resistances for the same classes of neurons as in **(C)**. (*N* = 9, 7, 9; *p* > 0.05 for all pairwise comparisons).

We performed whole-cell patch clamp recordings in CoPA neurons in 24–29 hpf embryos under current clamp conditions in order to characterize their intrinsic spiking properties and input resistances over this period of development. At 24–25 hpf, depolarizing current steps of increasing amplitude failed to elicit more than one action potential in the CoPA at the current onset (**Figures 1A,B**; *N* = 5/5). However, in embryos ≥26 hpf, CoPAs produced sustained firing throughout the duration of the current step and less current was needed to bring the neuron to threshold (**Figure 1A**; *N* = 9/9). A similar developmental maturation of firing has been shown in excitatory premotor spinal interneurons (Knogler et al., 2014). By 26 hpf, action potentials had large amplitudes and were always overshooting (**Figure 1A**; *N* = 9). The instantaneous spiking frequency during depolarizing current steps (4–36 pA; steps of 4 pA) was calculated for CoPA neurons in 24–25 hpf embryos vs. 26–28 hpf embryos to produce an F–I curve (**Figure 1B**; *N* = 5,9). All CoPAs ≤25 hpf fired singly with current injection whereas CoPAs from older embryos clearly increased their firing frequency with greater amounts of positive current. Though they were slightly less excitable at lower stimulus intensities, at higher stimulus intensities the response of CoPA neurons resembled that of CoSA neurons (**Figure 1B**; *N* = 9), which also have commissural ascending axons and are highly active at this age (Bernhardt et al., 1990; see later). A 32 pA current injection resulted in burst firing in all CoPA and CoSA neurons as well as in primary motoneurons and was used to compare average instantaneous firing frequencies across cell type (**Figure 1C**). The average firing frequencies for CoPAs, CoSAs, and motoneurons were not significantly different (69.4 ± 2.8, 76.1 ± 2.3, and 79.0 ± 3.3 Hz, respectively; *p* > 0.05 for all pairwise comparisons; *N* = 9, 5, 9). Embryonic motoneurons typically have input resistances in the 1–2 GΩ range (Saint-Amant and Drapeau, 2000; Tong and McDearmid, 2012; Knogler et al., 2014) and our recordings showed input resistances averaging 2.0 ± 0.4 GΩ for primary motoneurons and 3.2 ± 0.4 and 3.1 ± 0.1 GΩ for CoPAs and CoSAs, respectively, (**Figure 1D**; *p* > 0.05 for all pairwise comparisons; *N* = 9, 7, 9).

These results show that CoPA interneurons are undergoing a maturation of intrinsic properties subsequent to the onset of the touch response at 21 hpf and preceding the onset of swimming at 29 hpf (Saint-Amant and Drapeau, 1998). By 26 hpf, the threshold for action potentials has lowered and CoPAs have switched to a sustained firing mode during current injection. The similarity of intrinsic membrane properties between different cell types at this embryonic stage suggests that this maturation may occurring in many classes of spinal neurons during this developmental period (Knogler et al., 2014).

### EMBRYONIC CoPAs SHOW SPONTANEOUS LONG-LASTING GLYCINE-MEDIATED DEPOLARIZATIONS THAT SHUNTS EXCITATION

**FIGURE 2:**
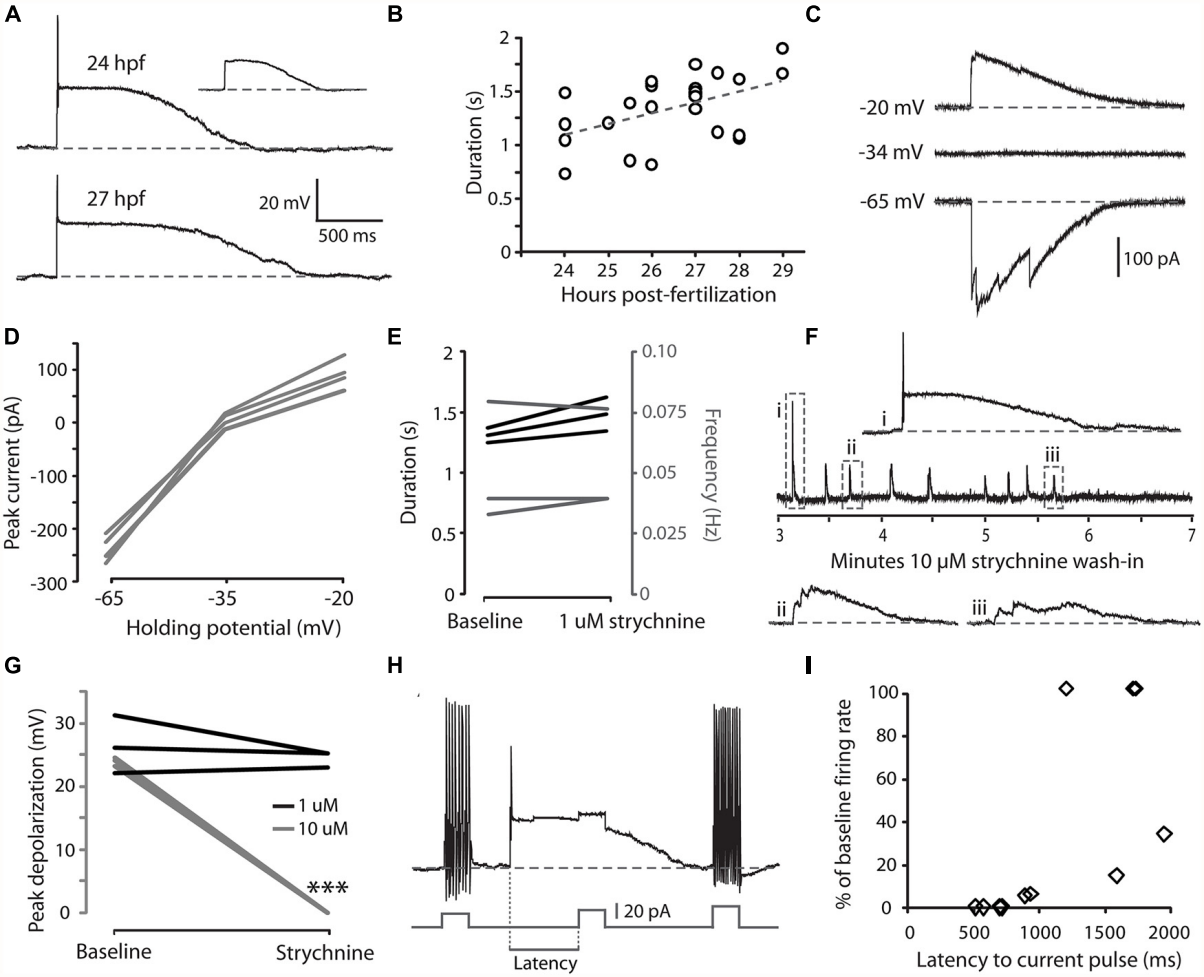
**Embryonic CoPAs show spontaneous activity in the form of a long-lasting depolarization that has low strychnine sensitivity and shunts excitation. (A)** Large upper trace, representative example of a whole-cell current-clamp recording of a spontaneous depolarizing event in a CoPA interneuron from a 24 hpf embryo that often triggered a small action potential at the onset. Baseline is shown as a dashed gray line for reference and resting membrane potential is –60 ± 5 mV in this and all subsequent current-clamp recordings. Inset upper right, example of a spontaneous depolarizing event in the same neuron that does not produce an action potential (scaled to 50%). Lower trace, a spontaneous depolarizing event recorded from an older 27 hpf embryo. **(B)** A significant positive correlation was found between embryonic age and the duration of depolarizing events in CoPAs. Pearson’s correlation coefficient *r* = 0.49 (*p* < 0.05; *N* = 25). The best linear fit is shown as a gray dashed line. **(C)** Representative whole-cell voltage-clamp recording from a CoPA neuron showing that the spontaneous currents are reversed at less negative holding potentials (–20 mV) compared to baseline (–65 mV) and that the net current is zero at –34 mV, the approximate chloride reversal potential. Timescale same as in **(A)**.**(D)** Average peak currents (not including initial spike) for CoPAs at three different holding potentials (*N* = 4). **(E)** Line graph showing that spontaneous depolarizing events do not change in duration (black lines) or frequency (gray lines) following addition of 1 μM strychnine (*p* > 0.05; *N* = 3). **(F)** Middle trace, a four-minute excerpt of a current-clamp recording of spontaneous CoPA activity in a 27.5 hpf embryo showing how 10 μM strychnine washing into the extracellular solution eliminates all spontaneous depolarizations over the course of several minutes. Events in dashed boxes i–iii are shown on an expanded timescale for clarity. Scale for i–iii same as in **(A)**. **(G)** Line graph showing that spontaneous depolarizing events are maintained in 1 μM strychnine (black lines; *p* > 0.05; *N* = 3) but completely lost in 10 μM strychnine (gray lines; ^∗∗∗^*p* < 0.001; Student’s *t*-test for each neuron pre- and post-drug application; *N* = 3). **(H)** An example of an injection of positive current in a CoPA from a 27 hpf embryo that normally results in sustained action potential firing at baseline (beginning and end of trace) but fails to elicit any action potentials during a spontaneous depolarizing event (middle of trace). Scale for upper trace same as in **(A)**, scale for current injection is shown. The measure of latency used for the subsequent is indicated. **(I)** Scatterplot of the reduction in firing frequency as a percentage of baseline when a positive current injection arrives during a spontaneous depolarizing event at various delays. *N* = 11 events from five embryos.

We have previously shown that CoPAs exhibit an activity pattern during the time window of spontaneous coiling behaviors that, unlike for premotor and motor neurons, is not modified during the transition from single to double coiling (Knogler et al., 2014). We examined the physiological properties of CoPAs in greater detail by single cell patch clamp recording. All current-clamp recordings from CoPA cells (*N* = 25/25) showed the presence of regular, spontaneous ∼ 25 mV amplitude depolarizations that were often sufficient to elicit a single action potential at the onset of the depolarization (**Figure 2A** and inset). In comparison to the spikes fired during current injection, this action potential had a smaller amplitude and was rarely overshooting (compare **Figures 2A** vs. **1A**). Despite the ability of these cells at ages ≥26 hpf to produce sustained firing of action potentials in response to depolarizing current injections (**Figure 1A**), multiple action potentials were never produced during spontaneous depolarizing events (**Figure 2A**; *N* = 25/25). These spontaneous depolarizations lasted more than one second at 24 hpf and this duration was found to increase in correlation with age (**Figure 2B**; e.g., average duration at 24 hpf = 1.11 ± 0.16 s, 27 hpf = 1.50 ± 0.06 s; Pearson’s correlation coefficient *r* = 0.49, *p* < 0.05). These durations are more than twice as long as for spontaneous activity patterns in other spinal interneurons and motoneurons at this age (Knogler et al., 2014). Occasional events were seen in CoPAs with durations longer than two seconds that resembled two overlapping events (data not shown; these events were not included in calculating duration averages in **Figure 2B**). Therefore, CoPAs showed what appeared to be unique, long-lasting shunting depolarization under physiological conditions.

We next investigated the nature of the CoPA depolarization. By 26 hpf, both glutamatergic and glycinergic signaling can be detected in the zebrafish spinal cord (Ali et al., 2000a; Saint-Amant and Drapeau, 2000, 2001; Pietri et al., 2009; Knogler et al., 2014) whereas GABAergic signaling does not play an important role (Saint-Amant and Drapeau, 2000; Buss and Drapeau, 2001). Glycine is depolarizing at this age due to a high intracellular chloride concentration (Saint-Amant and Drapeau, 2000) and can produce suprathreshold post-synaptic potentials depending on the reversal potential for chloride ions relative to the threshold for action potentials (Jean-Xavier et al., 2007). Glycinergic currents can be isolated from depolarizing glutamatergic currents pharmacologically with receptor antagonists or by holding the cell at the reversal potential for cations (approximately 0 mV) and anions (approximately –35 mV; Buss and Drapeau, 2001). Under voltage-clamp at resting membrane potentials (–65 mV), spontaneous currents in CoPAs were inward, whereas at less negative potentials (–20 mV), the direction of the currents reversed and when clamped at the chloride reversal potential there was no net flow of current (**Figures 2C,D**; *N* = 4). These results suggested that the spontaneous depolarizations seen in the CoPA were due to the flow of chloride ions and therefore we examined the effect of glycine receptor blockade on this activity.

Our initial attempts to block these depolarizing events in CoPA interneurons with 1 μM strychnine failed to do so nor to significantly alter the duration or frequency of these events (**Figure 2E**; *p* > 0.05; *N* = 3) despite the fact that this dose rapidly and effectively blocks the glycine-mediated depolarizations that are seen in motoneurons and other interneurons at this same age (Saint-Amant and Drapeau, 2000, 2001; Knogler et al., 2014). Previous studies have shown that different glycine receptor subunits may have different sensitivities to strychnine (Kuhse et al., 1990). We therefore tried a higher dose of 5–10 μM strychnine and saw that these depolarizing events were lost at this concentration (**Figures 2F,G**; *N* = 5). We believe that the effect of strychnine to reduce CoPA activity was *via* direct blockade of glycine receptors on the cell rather than the blockade of presynaptic inputs in the circuit based on two observations. First, the amplitude (not frequency) of CoPA depolarization decreased progressively when 10 μM strychnine was introduced (**Figure 2F**), suggestive of an increasing blockade of receptors. Secondly, as previously mentioned, 1 μM strychnine effectively blocks glycinergic currents in other cells whereas the glycine-mediated depolarizing events in CoPAs required a concentration of 10 μM strychnine for full blockade (**Figure 2G**; *p* < 0.001 for 10 μM strychnine; *N* = 6), in keeping with a different sensitivity of their respective receptors to strychnine. No effect on spontaneous depolarizing events was seen with 10 μM bicuculline, a blocker of GABA receptors (*N* = 2), or with 10 μM CNQX, a blocker of AMPA receptors (*N* = 2), therefore these depolarizing events appeared to be mediated by chloride currents through glycine receptors.

Glycine receptor-mediated depolarizing conductance may shunt excitation and functionally inhibit immature neurons (Jean-Xavier et al., 2007). In order to further examine the effect of these glycinergic depolarizations on action potential firing, we injected positive current at short latencies following the onset of a spontaneous depolarization and measured the rate of action potential firing compared to the same amount of current injection at baseline. An injection of depolarizing current that was sufficient to produce sustained firing at resting membrane potentials was far less effective at driving action potentials when applied during the first second of a spontaneous depolarizing event (**Figures 2H,I**; *N* = 11 events from five embryos), indicating that the CoPA is functionally inhibited by the depolarizing glycinergic conductance.

These results suggest that during the period of spontaneous behaviors, CoPAs receive glycinergic inputs that activate a chloride conductance leading to a long duration depolarizing event that is capable of shunting the membrane resistance and temporarily increasing the threshold for action potential firing. The long duration of spontaneous depolarizing events and lower sensitivity to strychnine compared to other interneurons and motoneurons at this age suggest that the CoPA may receive glycinergic inputs and/or express glycine receptors that are different from other spinal neurons.

### EMBRYONIC CoPAs HAVE SLOW GLYCINE-MEDIATED SYNAPTIC CURRENTS

**FIGURE 3:**
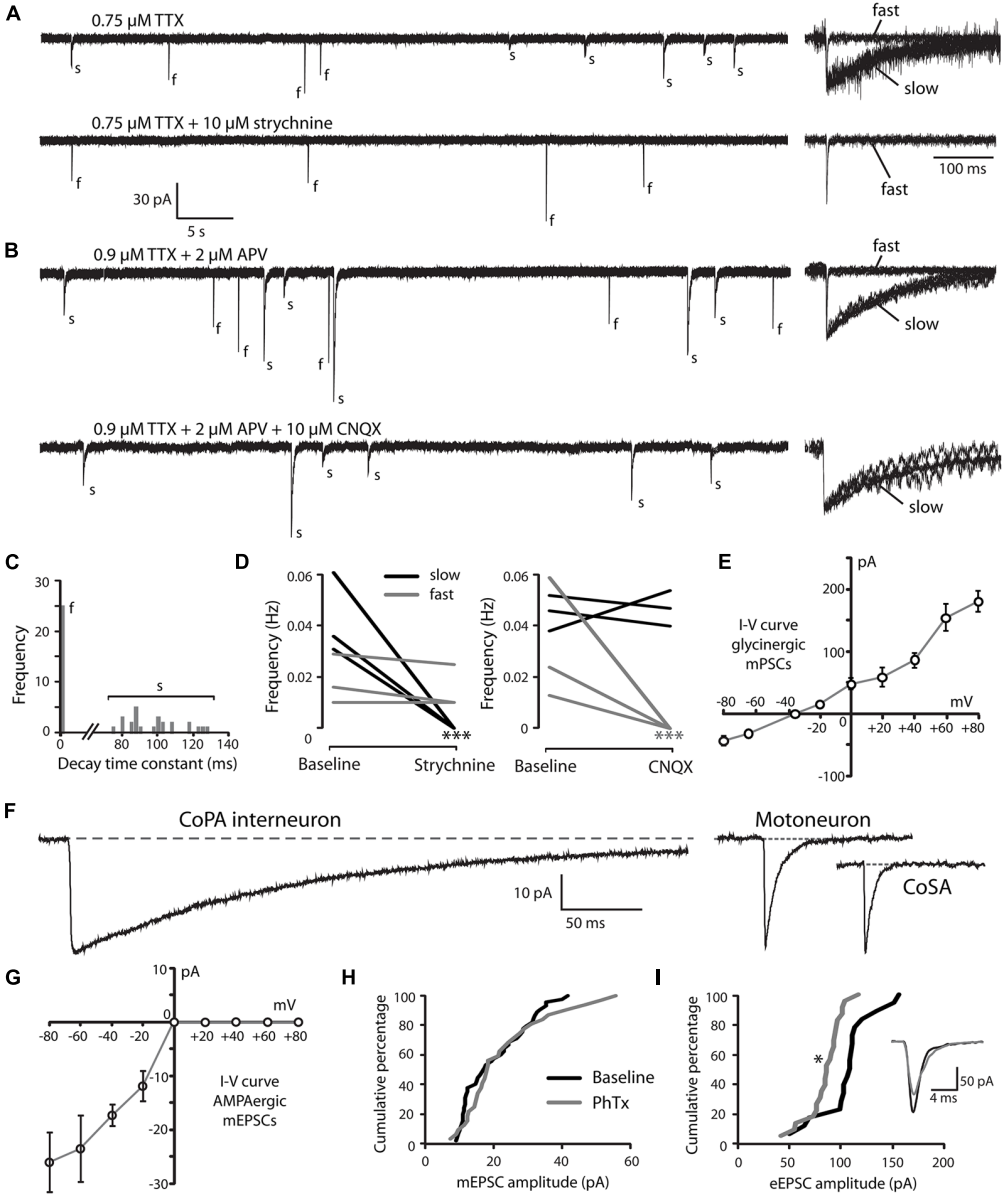
**Embryonic CoPAs have slow glycinergic miniature post-synaptic currents (mPSCs). (A)** Representative excerpt of a whole-cell voltage-clamp recording from a CoPA interneuron in a 29 hpf embryo showing the presence of two distinct categories of mPSCs in the presence of 0.75 μM tetrodotoxin (TTX) alone (upper trace, left) labeled “s” and “f” to denote slow and fast events, respectively. Following the additional wash-in of 10 μM strychnine (lower trace, left), slow events are lost but fast events remain, indicating that slow events are glycinergic. Insets at the right show the vertically scaled overlay of all events from the recording excerpt at the left on an expanded timescale. **(B)** Representative excerpt of a whole-cell voltage-clamp recording of mPSCs from a CoPA interneuron in a 28 hpf embryo in the presence of 0.9 μM TTX and 2 μM (2R)-amino-5-phosphonovaleric acid (APV; upper trace) with events labeled as in **(A)**. Following the additional wash-in of 10 μM CNQX (lower trace), fast events are lost but slow events remain, indicating that fast events are glutamatergic. Scale for traces and inset same as for **(A)**. **(C)** Frequency histogram of the first 50 events from the recording in TTX only in **(A)** showing a clear bimodal distribution of the decay time constants for mPSCs. **(D)** Left, line graph showing that the frequency of slow glycinergic mPSCs (black lines) is abolished with 10 μM strychnine (^∗∗∗^*p* < 0.001; for comparison of mini frequency from each neuron pre- and post-drug application) while fast glutamatergic mPSCs (gray lines) are unaffected (*p* > 0.20; *N* = 3). Right, line graph showing that fast mPSCs are abolished with 10 μM CNQX (^∗∗∗^*p* < 0.001; for comparison of mini frequency from each neuron pre- and post-drug application) while slow mPSC frequency is unaffected (*p* > 0.20; *N* = 3). **(E)** I–V curve of the average peak amplitude for glycinergic mPSCs as a function of membrane holding potential. **(F)** Representative example of an averaged glycinergic mPSC from a recording in a CoPA (left trace, *N* = 19 events), a motoneuron (middle trace, *N* = 30 events), and a CoSA neuron (right trace, *N* = 13 events) in a 29 hpf embryo in the presence of 1 μM TTX and 0.7 μM CNQX. See **Table 1** for quantification of mPSC properties. **(G)** I–V curve of the average peak amplitude for AMPAergic mPSCs as a function of membrane holding potential. See **Table 2** for quantification of mEPSC properties. **(H)** Cumulative histogram of AMPAergic mEPSC amplitudes at baseline (black trace) and following 10 μM PhTX treatment (gray trace; *p* > 0.05; *N* = 3). **(I)** Cumulative histogram of evoked AMPAergic EPSC amplitudes at baseline (black trace) and following 20 μM PhTX treatment (gray trace; ^∗^*p* < 0.05; Kolmorgorov-Smirnov test for distributions pre- and post-drug application; *N* = 3). Inset, overlay of averaged evoked AMPAergic EPSCs from one recording.

Developmental speeding of currents by subunit switching appears to be a general feature of vertebrate transmitter receptors, including those for glycine (reviewed by Takahashi, 2005). In order to examine the properties of glycine receptors in CoPA interneurons, we recorded miniature post-synaptic currents (mPSCs) in 26–29 hpf embryos. Recordings made in the presence of TTX to inhibit action potentials revealed the presence of depolarizing mPSCs with fast or slow rates of decay (category “s” and “f” events, respectively; **Figures 3A,B**; *N* = 3 for both). To confirm this categorization, a frequency histogram plot of all mPSCs for a cell clearly showed a bimodal, non-overlapping distribution of decay time constants separating fast from slow currents (**Figure 3C**). The mPSCs with slower kinetics were identified as glycinergic, as they were abolished by 10 μM strychnine (**Figures 3A,D**; *p* < 0.001; *N* = 3). The mPSCs with faster kinetics were identified as glutamatergic, as they were abolished by 5–10 μM CNQX (**Figures 3B,D**; *p* < 0.001; *N* = 3). We further confirmed that the slow currents were chloride based (glycinergic) by measuring the I-V curve at holding potentials ranging from –80 mV to +80 mV in 20 mV steps. As expected, glycinergic mPSC amplitudes decreased as holding potentials moved from –80 mV toward –35 mV, the approximate chloride reversal potential and did not show rectification (**Figure 3E**; *N* = 3). The frequency of both types of mPSCs was low (≤0.06 Hz; **Figures 3A,B,D**) therefore since the two populations could be easily resolved based on kinetics alone, some recordings omitted CNQX in order to collect both fast and slow mPSCs.

Glycinergic mPSCs were recorded from CoPA interneurons, motoneurons, and CoSA interneurons for comparison in 26–29 hpf embryos and their physiological properties are listed in **Table 1**. Glycinergic mPSCs in all cell types had similar amplitudes of approximately 30 pA (**Figure 3F**; *p* > 0.20 for all pairwise comparisons) but were present at twice the frequency in CoPAs than in motoneurons (mPSC frequency was lower and could not be accurately determined in CoSA recordings). Rise times were significantly slower in CoPAs than in MNs or CoSAs (*p* < 0.005) but the most striking difference was the order of magnitude longer decay time constant (τ) of glycinergic mPSCs in CoPAs compared to MNs and CoSAs (**Figure 3F**; *p* < 0.001). The decay time constant for CoPAs, nearly 100 ms, was similar to that observed for glycinergic mPSC and single channel kinetics in embryonic and adult Mauthner cells (Ali et al., 2000b; Hatta et al., 2001) and is within the range of values reported for rodent embryonic glycine receptors (Mangin et al., 2003). The decay time constants for CoSAs and motoneurons, in contrast, resembled the faster kinetics of glycinergic mPSCs in larvae and a subset of events in adult Mauthner cells (Ali et al., 2000b) as well as in P10–P20 rat motoneurons (Burzomato et al., 2004; Singer et al., 1998). The long decay time constant in CoPAs translated to a much larger charge transfer than in MNs and CoSAs (*p* < 0.001). No change in glycinergic mPSC parameters in CoPAs was seen with APV treatment (*N* = 4).

**Table 1:** Properties of glycinergic mPSCs in spinal neurons from 26 to 29 hpf embryos.

	CoPA	MN	CoSA
*N*	12	9	5
Age (hpf)	27.5 ± 0.2	27.6 ± 0.4	27.5 ± 0.5
Frequency (Hz)	0.043 ± 0.006^###^	0.020 ± 0.003	ND
Amplitude (pA)	31.1 ± 4.6	30.2 ± 2.7	31.5 ± 4.5
10–90% rise time (ms)	1.31 ± 0.13**	0.80 ± 0.06	0.65 ± 0.08
Decay time constant (ms)	90.5 ± 5.7***	7.56 ± 0.70	5.16 ± 0.52
Charge transfer (nC)	2.70 ± 0.38***	0.25 ± 0.03	0.16 ± 0.02

^#^Student’s *t*-test; *Tukey’s *post hoc* test; ND = not determined.

Based on these mPSC properties and the low strychnine sensitivity of spontaneous currents, CoPAs appear to express different glycine receptors than CoSAs and motoneurons. The slow kinetics of glycine receptors in embryonic CoPA interneurons likely contribute to the long duration of their depolarizations following receptor activation and the resulting shunting of action potential firing.

### CoPA INTERNEURONS EXPRESS PhTX-INSENSITIVE AMPA RECEPTORS THAT MEDIATE RECTIFYING GLUTAMATERGIC mEPSCs

Our recordings of miniature PSCs in CoPA in the presence of TTX and APV, an NMDA receptor antagonist, revealed the presence of fast, CNQX-sensitive AMPAergic currents that were easily distinguishable from slow glycinergic mPSCs (**Figures 3A,B**). These fast currents were confirmed to be non-selective cationic (glutamatergic) by holding the membrane potential at the approximate reversal potentials for cations (0 mV; **Figure 3G**). AMPAergic mEPSCs were rare at 24 hpf but increased rapidly in amplitude and frequency over development, therefore recordings were made in 26–29 hpf embryos to obtain a sufficient number of events. The properties of AMPAergic mEPSCs at this age are summarized in **Table 2**. (In both motoneurons and CoSAs at this age the frequency of mEPSCs was far lower, therefore we did not include these cells for comparison.) On average, the amplitude and frequency of AMPAergic mEPSCs were similar to glycinergic mPSCs, but the kinetics of glutamatergic mEPSCs were far faster than those of glycinergic events in terms of both rise time and decay, leading to two orders of magnitude lower average transfer of charge per event (**Figures 3A,B**). In addition, unlike glycinergic mPSCs whose properties were stable over the period of 26–29 hpf, the amplitude of AMPAergic mEPSCs increased over this short, 4-hour window of time with a high and statistically significant correlation between amplitude and embryo age (Spearman rank rho = 0.76, *p* < 0.05; *N* = 9). AMPAergic mEPSCs also showed a trend toward increased frequency with embryo age although this correlation was not statistically significant (Pearson’s correlation *r* = 0.51; *p* = 0.08; *N* = 9).

**Table 2:** Properties of AMPAergic mPSCs in CoPA interneurons from 26 to 29 hpf embryos.

	CoPA interneuron
*N*	9
Age (hpf)	27.4 ± 0.3
Frequency (Hz)	0.038 ± 0.010
Amplitude (pA)	25.7 ± 5.5
10–90% rise time (ms)	0.41 ± 0.020
Decay time constant (ms)	1.15 ± 0.040
Charge transfer (nC)	0.040 ± 0.009

AMPAergic I-V curves were obtained by recording AMPAergic mEPSCs at holding potentials ranging from –80 mV to +80 mV in 20 mV steps in order to determine the degree of rectification. As expected, AMPAergic mEPSC amplitudes decreased as holding potentials moved from –80 mV toward 0 mV, the approximate cationic reversal potential (**Figure 3G**; *N* = 3). However, no outward-going AMPAergic mEPSCs were seen beyond the cationic reversal potential, despite the large driving force present at +80 mV. Despite having a larger signal to noise ratio, evoked AMPAergic EPSCs onto CoPAs also failed to reverse at positive holding potentials (*N* = 3; data not shown), demonstrating a strong inward rectification of glutamatergic currents, consistent with the presence of calcium-permeable AMPARs that should be sensitive to external polyamine block (Man, 2011). We examined the sensitivity of these receptors to external polyamine block by applying 10 μM philanthotoxin 343 (PhTX) to our recordings of AMPAergic mEPSCs from CoPAs. No consistent change in mEPSC amplitudes was seen following PhTX treatment (**Figure 3H**; *N* = 3; *p* > 0.20). In order to ensure adequate receptor blockade, we applied 20 μM PhTX in combination with repeated activation of the receptors and saw that the amplitude of evoked glutamatergic events in CoPAs was significantly reduced by 23 ± 7% (**Figure 3I**; *N* = 3; *p* < 0.05).

These results suggest that glutamatergic inputs to CoPA interneurons are maturing rapidly over the period of development preceding swimming as evidenced by the increase in both amplitude and frequency of AMPAergic mEPSCs. Post-synaptic glutamatergic signaling in CoPAs appears to be mediated by strongly rectifying but mostly PhTX-insensitive AMPARs that may belong to a recently described functional class of AMPARs seen in the developing retina and hippocampus (see Discussion for details). Thus, both glutamatergic and glycinergic synaptic currents are atypical in the CoPA neurons and may be related to their unusual physiological properties.

### EMBRYONIC CoPAs ARE NOT PART OF THE NETWORK DRIVING IPSILATERAL COILS AND ARE SHUNTED BY GLYCINERGIC INHIBITION DURING CONTRALATERAL COILS

Having examined the properties of spontaneous synaptic inputs to CoPAs, we were next interested in looking at glycinergic and glutamatergic inputs to CoPAs in a behavioral context. The synaptic inputs to motoneurons during both spontaneous and evoked embryonic behaviors have been described in several studies (Saint-Amant and Drapeau, 2000, 2001; Pietri et al., 2009; Knogler et al., 2014). Briefly, spontaneous single coils are unilateral trunk contractions driven by electrically coupled ipsilateral cells generating low-amplitude depolarizing periodic inward currents (PICs) in motoneurons (Saint-Amant and Drapeau, 2000). During embryonic development, nascent glutamatergic inputs give rise to double coils, a transient developmental behavior that precedes swimming (Knogler et al., 2014). When the trunk is contracting in a coil, motoneurons contralateral to the coiling side receive large amplitude depolarizing glycinergic synaptic bursts (SBs), but these inputs do not drive the behavior itself (Saint-Amant and Drapeau, 2001). We can therefore interpret the fictive behavior in the paralyzed animal electrical by knowing that electrical depolarizations in motoneurons drives ipsilateral coiling while glycinergic bursts are the physiological correlate of contralateral coiling (Saint-Amant and Drapeau, 2001; Pietri et al., 2009; Knogler et al., 2014).

**FIGURE 4:**
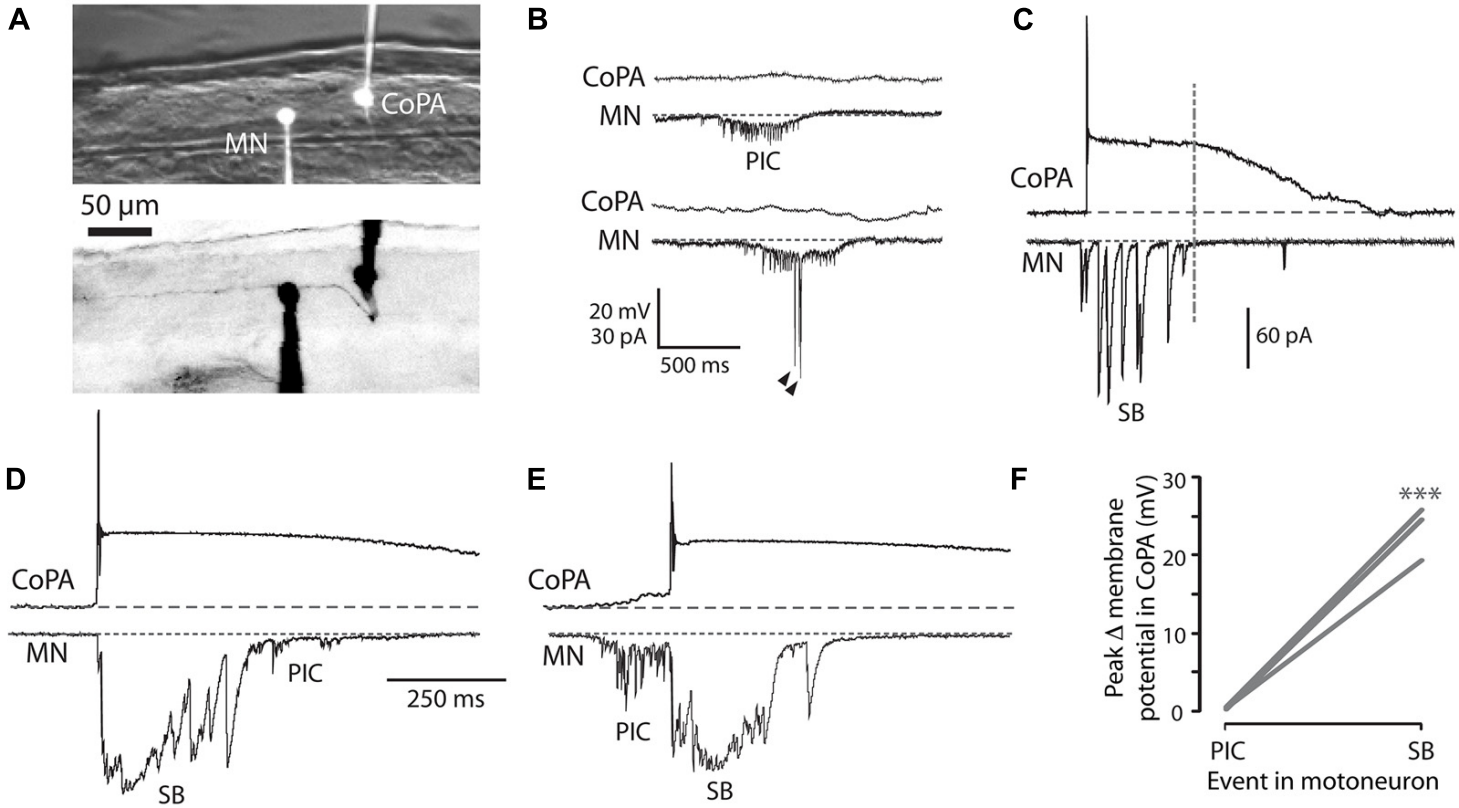
**Embryonic CoPAs are inactive during fictive ipsilateral coils and are depolarized by glycinergic inputs during fictive contralateral coils. (A)** Image of filled neurons from a simultaneous whole-cell recording of a CoPA and ipsilateral MN. The upper image shows the filled cell bodies in fluorescence against the spinal cord in brightfield. The lower image shows the inverted fluorescent image of rhodamine-filled ipsilateral cell bodies with the ascending contralateral CoPA axon in focus spanning several somites (the ventral MN axon is obscured by the recording electrode). Rostral is to the left and dorsal is to the top. **(B)** Simultaneous whole-cell recordings from a 26 hpf embryo of activity in a CoPA (current clamp, top trace in all pairs) and a MN (voltage-clamp, bottom trace in all pairs) during a spontaneous fictive ipsilateral single coil. The CoPA is inactive during a gap junction-driven current (periodic inward current, PIC) in the ipsilateral MN. Holding potential of MN was –65 mV and baseline is shown as a dotted gray line. Arrowheads in MN trace denote large glutamatergic peaks. **(C)** Depolarizing glycinergic events in CoPAs coincide with glycinergic synaptic bursts (SBs) in ipsilateral MNs during a spontaneous fictive contralateral single coil. Scale same as in (B) but note different vertical scale for MN trace. A vertical dashed line marks the end of the SB in the MN trace. **(D)** A depolarizing glycinergic event in a CoPA during a spontaneous fictive double coil (synaptic burst, SB, followed by PIC) in a MN resembles the activity seen during a single coil and coincides with the glycinergic portion of the mixed event in the MN. Scale same as in **(C)** but note different time scale. **(E)** Same conditions as **(D)** but here for a fictive double coil beginning on the ipsilateral side (PIC preceding SB). All recordings are from the same pair of neurons pictured in **(A)**. **(F)** Comparison of the average change in membrane potential for CoPAs during each type of activity in the MN (^∗∗∗^*p* < 0.001; Mann-Whitney *U*-test for each neuron between event types).

We performed simultaneous whole-cell recordings from ipsilateral CoPAs and motoneurons in order to correlate activity in the CoPA with fictive spontaneous single and double coiling behavior (**Figure 4A**; *N* = 3). Unlike the majority of other interneurons at this age, CoPAs were inactive during fictive ipsilateral single coils, as evidenced by a complete absence of activity during PICs (with or without coinciding glutamatergic events) in the motoneuron (**Figures 4B,F**). Glycine-mediated depolarizations of CoPAs were, however, synchronized with glycinergic synaptic bursts in the ipsilateral motoneuron (**Figures 4C,F**), indicative of a fictive contralateral coil. Interestingly, the duration of the depolarization in CoPAs far outlasted that of the inputs to motoneurons. Upon closer examination, the duration of the maximal depolarization in the CoPA appeared to overlap with the duration of the synaptic burst in the MN (**Figure 4C**, dashed vertical line), suggesting that their glycinergic inputs may be similar but have distinct postsynaptic effects due to the far slower kinetics of CoPA glycine receptors. The occurrence of spontaneous fictive double coils as represented by mixed electrical (PIC) and glycinergic events (SB) in the motoneuron (Knogler et al., 2014) did not correlate with any new activity pattern in the CoPA (**Figures 4D,E**). Once again, no activity was seen in the CoPA during the electrical component of the mixed event in the motoneuron (**Figures 4E,F**).

These simultaneous recordings suggest that the glycinergic depolarizing events in the CoPA occur during a contralateral coiling event. Since the blockade of glycinergic signaling at this age does not block coiling behavior (Saint-Amant and Drapeau, 2000) and in fact may lead to multiple coils (Knogler et al., 2014), the CoPA is unlikely to be driving this spontaneous behavior. These results also demonstrate that the CoPA is not a part of the electrically coupled ipsilateral network, further suggesting that the CoPA does not play a key role in driving spontaneous single coils. Finally, the inputs onto the CoPA are not different during a double coil than during a single coil, supporting our previous hypothesis (Knogler et al., 2014) that this cell is not part of the circuit driving double coiling behavior.

### EMBRYONIC CoPAs RECEIVE BRIEF GLUTAMATERGIC EXCITATION FOLLOWED BY LONG LASTING, SHUNTING GLYCINERGIC INPUTS IN RESPONSE TO TOUCH

Having shown that CoPAs are not likely to contribute to spontaneous coiling behaviors, we next examined their activity during touch-evoked coiling behavior. CoPAs are known to receive glutamatergic followed by glycinergic inputs in response to a touch stimulus at 24 hpf (Pietri et al., 2009). The depolarizing glycinergic inputs are presumed to inhibit activation of the CoPA but the effect of these currents on membrane potential and action potential firing has not been shown.

**FIGURE 5:**
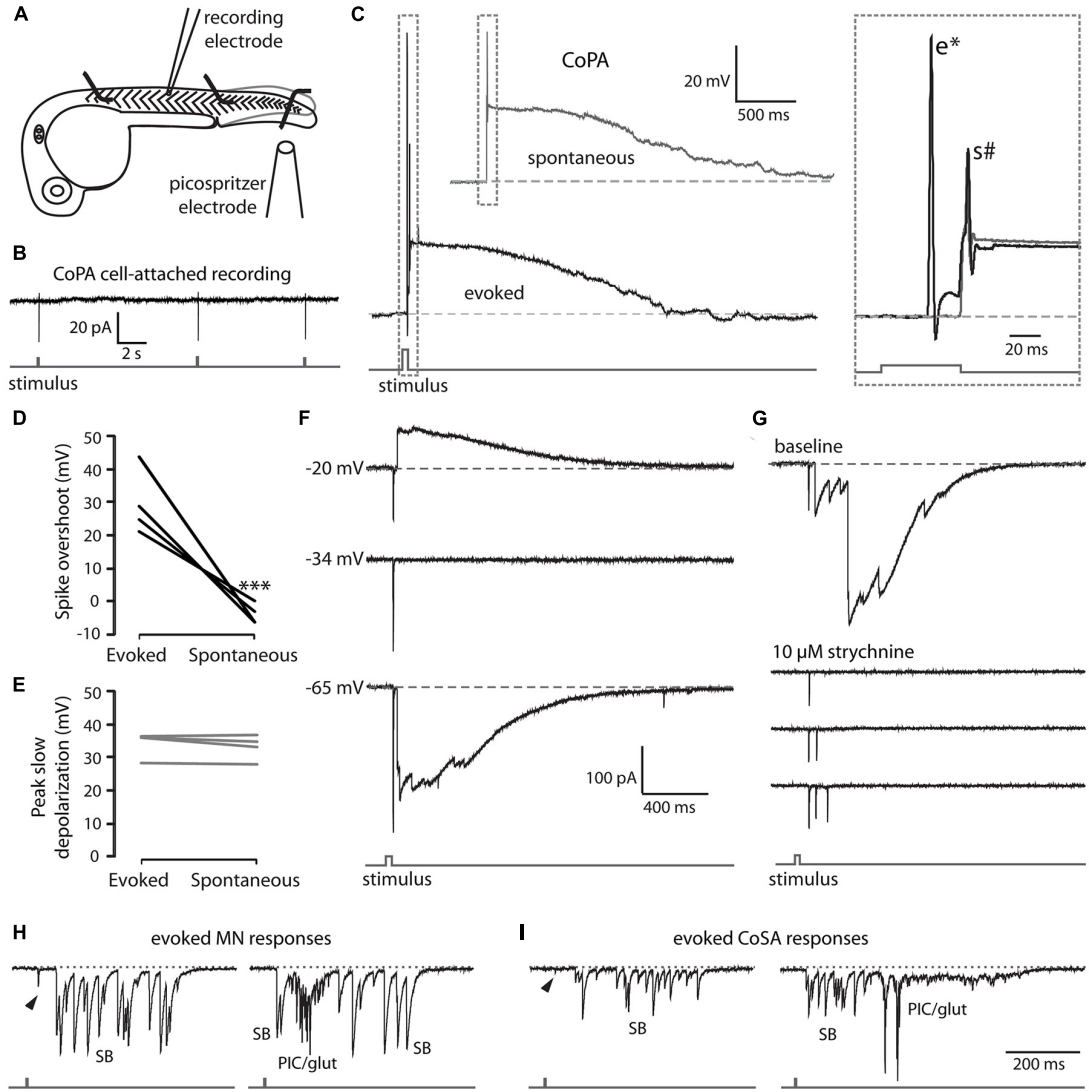
**EmbryonicCoPAs receive brief glutamatergic excitation then long lasting, shunting glycinergic inputs in response to touch. (A)** Cartoon depicting the experimental set-up for recording touch-evoked responses. **(B)** Representative cell-attached recording of single spikes consistently elicited in CoPAs (upper trace) in response to touch stimuli (lower trace). No spontaneous spikes were seen in the absence of stimuli. *N* = 3. **(C)** Representative current-clamp recordings of a spontaneous event (upper trace, gray) and an evoked event (middle trace, black, and lower stimulus trace, gray) in a 26.5 hpf embryo. Inset, expanded view of the overlapping traces to show the presence of a large amplitude action potential (e*) occurring in the evoked event only and a second smaller action potential (s#) occurring in both events. **(D)** Comparison of the average overshoot reached by the first spike in an evoked vs. spontaneous event (^∗∗∗^*p* < 0.001; Student’s *t*-test for each neuron between event types). **(E)** Comparison of the average peak slow depolarization during an evoked vs. spontaneous event (*p* > 0.20). **(F)** Voltage-clamp recording from the same neuron as in C showing that the evoked response is mediated by two different synaptic inputs: a short-latency glutamatergic EPSP that decreases in amplitude at less negative holding potentials (–20 mV) but does not reverse, and a later, long duration glycinergic input that reverses beyond –34 mV. **(G)** Voltage-clamp CoPA recording from a 26 hpf embryo showing that the wash-in in 10 μM strychnine selectively blocks the glycinergic currents but leaves the short latency, fast glutamatergic currents unaffected. Scale same as for **(F)**. **(H,I)** Voltage-clamp recordings of synaptic currents in a motoneuron **(H)** and a CoSA **(I)** evoked by an ipsilateral touch stimuli in a 27.5 hpf embryo. Scale same as in **(F)**. Glycinergic synaptic bursts are labeled SB and coincident electrical PICs and glutamatergic currents are labeled PIC/glut.

In order to characterize the spiking of CoPA interneurons following a touch stimulus, we performed electrophysiological recordings from CoPAs while squirting water onto the ipsilateral tail of the embryo to displace it slightly, evoking a touch response (**Figure 5A**). In cell-attached mode, the stimulus robustly elicited one spike in the CoPA but otherwise no spikes were seen spontaneously in any recordings (**Figure 5B**; *N* = 3). Under current-clamp conditions, the CoPA produced one or two action potentials then remained depolarized for one to two seconds following the stimulus (**Figure 5C** lower trace and inset; *N* = 4 embryos). The response was very similar to that seen during spontaneous events occurring between stimuli except that in addition to the small amplitude action potential seen during both spontaneous and evoked events, the evoked response showed a short latency, overshooting action potential with a much larger amplitude (**Figure 5C** and inset **Figure 5D**). The peak amplitude of the slow, long duration depolarization mediated by glycine was indistinguishable between evoked and spontaneous events (**Figure 5E**).

We hypothesized that the first action potential seen in the CoPA following the stimulus was a fast suprathreshold response mediated by a strong glutamatergic input as Pietri et al. (2009) have shown that CoPAs receive short latency glutamate-driven cationic currents followed by large glycine-driven chloride currents following an ipsilateral touch stimulus. We confirmed that the short latency (<20 ms) currents were cationic and remained inward at holding potentials up to –20 mV while the later arriving, larger currents were confirmed to be anionic currents as they disappeared at the chloride reversal potential (approximately –34 mV) and were reversed at –20 mV (**Figure 5F**). Wash-in of 10 μM strychnine abolished the glycine-driven chloride currents for evoked and spontaneous events while leaving evoked cationic currents unaffected (**Figure 5G**; *N* = 2). The blockade of glycinergic signaling also revealed that CoPA interneurons often received multiple glutamatergic inputs following the stimulus that would normally be occluded by the large amplitude glycinergic chloride currents (**Figure 5G**), suggesting that the arrival of shunting glycinergic inputs prevent sustained action potential firing even in the presence of multiple strong excitatory inputs. The additional glutamatergic EPSCs may be due to the activation of other RB neurons further away that contact the same CoPA.

These findings show that CoPAs fire a large, overshooting action potential triggered by short latency excitation from the glutamatergic RB in response to touch. Activation is followed quickly by the arrival of glycinergic inputs that produce a shunting response identical to that seen during a spontaneous coil, suggesting that CoPA is functionally inhibited in the same way during spontaneous and ongoing self-generated movement. These results further indicate that the response of the CoPA to glutamatergic inputs must be robust in order to produce this first, large amplitude action potential prior to the arrival of the shunting glycinergic inputs.

### OTHER CLASSES OF EMBRYONIC SPINAL NEURONS SHOW DIFFERENT ACTIVITY PATTERNS DURING SPONTANEOUS AND TOUCH-EVOKED ACTIVITY

In order to better understand how the activity of CoPAs differs from that of other cells, we recorded the responses in other spinal neuron classes at rest and following a touch stimulus in 24–29 hpf embryos. Following an ipsilateral touch stimulus normally producing a contralateral trunk contraction, motoneurons received a burst of depolarizing glycinergic inputs with a latency of several tens of milliseconds, (**Figure 5H**; *N* = 3) as previously shown at 24 hpf (Pietri et al., 2009). The glycinergic burst was capable of producing action potentials under current-clamp conditions (not shown) and was sometimes preceded by a small glutamatergic event (**Figure 5H**, arrowhead) with a shorter latency, but these inputs were insufficient to drive an action potential. A single touch response sometimes elicited up to three fictive alternating coils, as indicated by the sub-second succession of a glycinergic burst (SB) followed by an electrical depolarization (PIC) then another SB in the motoneuron recording (**Figure 5H**, right). Spontaneously occurring events strongly resembled evoked responses except that the spontaneous events never showed short latency glutamatergic events preceding the glycinergic burst (Knogler et al., 2014).

As previously discussed, CoSA interneurons resemble CoPAs morphologically (see **Figure 1B**) and it has been proposed that CoSAs may also play the role of sensory interneuron in the spinal cord (Hale et al., 2001; Liao and Fetcho, 2008). We recorded from embryonic CoSAs that were identified by their small, dorsal soma, ascending commissural axon, and lack of dendrites at this age compared to CoPAs (Bernhardt et al., 1990). Although CoSAs are a heterogeneous class of neurons in terms of their neurotransmitter identity, only glutamatergic CoSAs have been shown to be present in the spinal cord at this early stage (Satou et al., 2012). Our recordings showed that CoSAs were highly active with spontaneous and evoked activity patterns resembling motoneurons (**Figure 5I**) and also like motoneurons produced several spikes in cell-attached recordings both in response to touch stimuli and spontaneously (data not shown). A touch stimulus evoked a depolarizing glycinergic synaptic burst in ipsilateral CoSAs with a similar latency as in motoneurons and sometimes consisting of two or more additional fictive alternating coils (**Figure 5I**; *N* = 5). These evoked events were also sometimes preceded by small, short latency glutamatergic peaks (**Figure 5I**; arrowhead), which like in motoneurons were not sufficiently large to drive an action potential. Recordings (*N* = 7) of spontaneous activity from CoSAs showed activity patterns strongly resembling evoked responses (data not shown). These data suggest that CoSAs are part of the embryonic motor circuit active during spontaneous coiling behaviors but do not act as sensory interneurons at this age.

Finally, we also recorded from several embryonic CoBL neurons which at this stage are identifiable by their small dorsal soma and their unique bifurcating commissural axon that has both a major ascending and descending branch (Bernhardt et al., 1990). Our recordings failed to show any evidence of electrical depolarizations in CoBLs (*N* = 12). In fact, apart from two recordings in 28–29 hpf embryos, the majority of recordings (*N* = 10/12) also showed no evidence of synaptic glycinergic bursts and CoBLs never fired spontaneous action potentials. These findings indicated that CoBLs were not active during coiling behaviors and recordings during touch-evoked coiling supported these results (*N* = 2).

Our recordings from other commissural interneurons and motoneurons revealed two main patterns of activity. Spontaneous and evoked activity of motoneurons and CoSAs revealed depolarizing electrical as well as glycinergic events, demonstrating that these cell types participate in coiling behaviors. Both types of activity were capable of eliciting action potentials in these cells, particularly at later stages. In contrast, CoBLs did not fire action potentials during spontaneous or evoked activity, suggesting that despite having extended long axons by this age these cell types do not participate in coiling behaviors. These results suggest that CoSAs, the only other commissural interneuron class active at this age, participate in motor rather than sensory circuits and highlight the importance of the CoPA in relaying somatosensory input contralaterally during evoked embryonic behaviors.

### CoPAs RECEIVE RHYTHMIC INHIBITION IN PHASE WITH EXCITATION TO IPSILATERAL MNs DURING BURST SWIMMING EPISODES

The latest-appearing embryonic zebrafish behavior is swimming, which begins around 28 hpf. Swimming can be elicited at this age by a touch stimulus to the tail, or, to a lesser degree, to the head and may also appear spontaneously (Saint-Amant and Drapeau, 1998). During immature burst swimming in zebrafish, motoneurons receive tonic depolarization composed of both chloride and cationic conductances in addition to rhythmic excitatory post-synaptic currents that are capable of driving action potentials in the motoneuron throughout the duration of the behavior (Buss and Drapeau, 2001). This motoneuron activity drives ipsilateral muscle contractions while the contralateral motoneurons receive excitation out of phase, thus driving alternating contractions of the trunk as seen during free swimming behavior (Masino and Fetcho, 2005; McDearmid and Drapeau, 2006).

**FIGURE 6:**
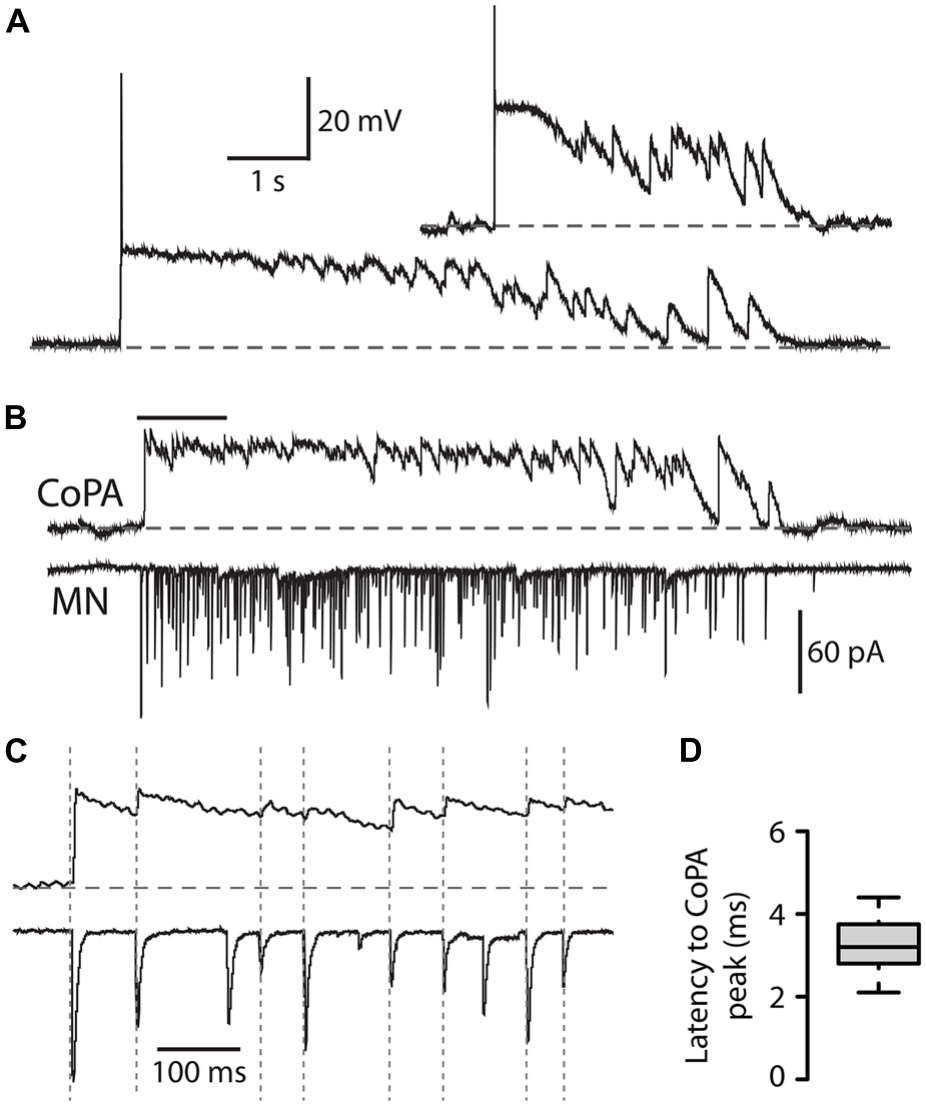
**Commissural primary ascending interneurons receive rhythmic inhibition in phase with excitation to ipsilateral MNs during burst swimming episodes. (A)** Two examples of activity patterns in CoPAs from embryos at 27–29 hpf that have longer durations than fictive coiling events and whose durations resemble those of bouts of immature swimming at this age. **(B)** Example of a simultaneous whole-cell recording from a 29 hpf embryo of activity in a CoPA (in current clamp) and an ipsilateral motoneuron (in voltage-clamp) during a spontaneous bout of fictive burst swimming. Scale for CoPA trace same as for **(A)**, vertical scale shown for MN trace. **(C)** Ten times horizontally expanded view of the burst from the indicated region in (B). Dotted vertical lines indicate local maxima (peaks) in the CoPA trace that correlate with MN peaks. **(D)** Quantification of the latency between peaks in the CoPA trace relative to peaks in the MN trace. *N* = 20 peaks from one event.

We recorded from CoPAs in 28–29 hpf embryos in the absence of touch stimuli in order to observe their activity patterns during spontaneous bouts of fictive burst swimming. Occasionally, an activity pattern was seen that we interpreted to be an episode of fictive immature burst swimming due to the unusually long duration (>3 s) of the activity (**Figure 6A**; *N* = 3 events in three embryos). This activity strongly resembled a longer duration version of the glycine-mediated depolarization seen during a coiling event in the CoPA (compare **Figures 6A and 2A**). Consistent with the activity pattern seen during coiling, no more than one small action potential at the onset was ever produced and no fast EPSPs were seen on top of the depolarization, suggesting that no glutamatergic inputs arrived to CoPAs during the period of swimming or that these inputs were not visible due to shunting.

In order to confirm that these observations of CoPA activity were indeed correlated with swimming activity, we obtained a simultaneous whole-cell recording from a CoPA interneuron and ipsilateral motoneuron in a 29 hpf embryo during which a bout of spontaneous fictive swimming occurred (**Figure 6B**). The CoPA showed a long duration depolarization that peaked at the chloride reversal potential and resembled the long duration events recorded in individual CoPAs (**Figure 6A**). The CoPA activity persisted for the duration of the excitatory inputs to the motoneuron and distinct peaks could be seen during the depolarization that were in phase with excitatory inputs to the ipsilateral motoneuron (**Figure 6C**). In this recording the peaks in the CoPA depolarization consistently followed the peaks in the MN excitation by on average 3.3 ± 0.2 ms (**Figure 6D**), a small fraction of the 30–100 ms interval between peaks. Due to the rarity of these spontaneous swimming events we were unable to confirm the identity of the currents by changing the reversal potential or with pharmacological receptor antagonists but the depolarization in the CoPA clamped the membrane potential near the chloride reversal potential and had slow decay kinetics, strongly suggesting that these were mediated by chloride and not cationic currents.

These results show that CoPAs receive rhythmic glycinergic inputs in phase with excitation to the ipsilateral motoneuron during swimming. The activity patterns in CoPAs during coiling and swimming are very similar apart from their durations, suggesting that the same type of presynaptic glycinergic neurons might mediate functional inhibition for these different behaviors. These findings support the hypothesis that CoPAs are actively inhibited by corollary discharge during different types of self-generated movement in the embryo.

## DISCUSSION

The goal of this study was to examine the intrinsic and synaptic properties of sensory CoPA interneurons during different embryonic behaviors in an effort to understand how these neurons might play a specialized role in gating activation of the somatosensory pathway during self-generated movements in the zebrafish embryo. We show that the synaptic properties of embryonic CoPAs are highly tuned for gating sensory information in order to ensure the appropriate activation of the touch response. Embryonic CoPAs receive shunting glycinergic depolarization mediated by slowly deactivating glycine receptors during contralateral spinal cord activation. We propose that this corollary discharge prevents inappropriate activation of sensorimotor pathways during a period in which the zebrafish exhibits high levels of spontaneous locomotor activity. In response to touch, fast glutamatergic input from RB sensory neurons arrives prior to glycine-mediated shunting and allows the brief, strong AMPAergic activation of CoPAs necessary to propagate and amplify excitation in the touch reflex pathway. These results further our understanding of how sensory processing is modulated by motor patterns in the vertebrate spinal cord.

### SHUNTING OF EMBRYONIC CoPA INTERNEURONS PREVENTS INAPPROPRIATE ACTIVATION OF THE TOUCH REFLEX DURING SPONTANEOUS OR ONGOING EVOKED BEHAVIORS

The mechanosensitive neurites of RB somatosensory neurons form an extensive network of coverage along the surface of the zebrafish trunk to mediate responses to touch or noxious stimuli. A single action potential in a single RB is sufficient to activate an escape behavior at 24 hpf (Douglass et al., 2008), which poses a challenge for the sensorimotor circuit to balance the need for a low rate of false activation under baseline conditions with robust activation when a real stimulus is present. As RBs may be activated and fire during ongoing behaviors such as coiling and swimming, there must be safety measures in place to inhibit ongoing pathway activation. Mechanisms such as presynaptic inhibition of sensory afferents have been observed in the vertebrate spinal cord that could contribute to inactivating RBs during ongoing mechanosensation (reviewed by Rudomin and Schmidt, 1999), although this remains to be examined in zebrafish RBs. Regardless of presynaptic RB firing, our results show that the inhibition of CoPA sensory interneurons provides a sufficiently strong gate to prevent inappropriate activation of the touch reflex.

Embryonic CoPAs show spontaneous patterns of activity in the form of one to two second-long depolarizations that outlast those seen in other spinal neurons at this age (Knogler et al., 2014). We have now shown that this is due to a large, glycine receptor-mediated chloride conductance that effectively shunts the membrane resistance and raises the threshold for action potential firing. In other cells such as CoSAs and motoneurons, similar glycinergic inputs excite cells and often lead to action potential firing. The dual nature of depolarizing glycinergic (or GABAergic) inputs in the developing nervous system has been widely reported and is known to depend on cellular properties such as resting membrane potential, intracellular chloride concentration, as well as the location of the inhibitory synapses on the neuron (Ben-Ari, 2002; Jean-Xavier et al., 2007). In our study, the efficacy of glycinergic PSCs in shunting activity in CoPAs more than in other cells, even given similar presynaptic inputs, appears to be due in large part to the presence of glycine receptors with slow kinetics in CoPAs. Our results suggest that the long decay phase of glycinergic currents in CoPAs results in effective and long-lasting shunting during spontaneous activity as well as during the ongoing activity of evoked behaviors such as swimming. It should be noted however, that these electrophysiological recordings were performed in paralyzed animals, therefore we cannot exclude the possibility that the activity of spinal sensory neurons (such as RBs) and interneurons could be different when the muscles are physically contracting.

The embryonic mammalian α2 glycine receptor subunit imparts slow decay time constants on homomeric α2 and heteromeric α2β glycine receptors (for reviews see Legendre, 2001; Lynch, 2004, 2009). We observed that the glycinergic mPSCs of CoPAs exhibit similarly slow kinetics, suggesting that glycine receptors contain α2 or similar (α4a) subunits. Teleost reticulospinal Mauthner cells exhibit glycine-mediated synaptic and single channel currents with similarly slow kinetics (Ali et al., 2000b; Hatta et al., 2001), an interesting comparison given that these cells are known to be specialized for touch-evoked behaviors (Eaton et al., 1977). The slow kinetics of these currents, particularly early in development when glycine is depolarizing, could ensure that Mauthner cells and CoPAs are adequately shunted during ongoing activity despite relatively few inputs at early stages (Ali et al., 2000b). Motoneurons and CoSAs, in contrast, showed fast glycinergic mPSCs that likely reflect the activation glycine receptors with faster α1 subunits. In addition to this difference in kinetics, a higher concentration of strychnine was required to block glycinergic currents in CoPAs in our experiments compared to other embryonic spinal neurons including Mauthner cells (Ali et al., 2000b; Saint-Amant and Drapeau, 2000). One glycine receptor subunit found to date only in neonatal mammalian neurons, α2^∗^, shows a significantly reduced affinity for strychnine as a ligand that has been linked to a difference in one amino acid residue from the more common embryonic α2 subunit (Becker et al., 1988; Kuhse et al., 1990). As a result, homomeric α2^∗^ receptors have a 500-fold lower sensitivity to strychnine than α2 receptors and show only a very small reduction in current in the presence of 1 μM strychnine (Kuhse et al., 1990; Han et al., 2004). Our results raise the possibility that CoPAs express α2^∗^-containing glycine receptors whereas other embryonic spinal neurons are more likely to express the common α2 or α1 subunit. Interestingly, a subset of slow glycinergic currents in adult Mauthner cells was also shown to be less sensitive to strychnine (Hatta et al., 2001). It will be interesting to determine if these slow subunits in CoPAs undergo developmental speeding by replacement with faster subunits or if slow currents persist throughout adulthood.

The identity of the presynaptic glycinergic neurons in zebrafish that provide corollary discharge onto CoPAs is not currently known. In *xenopus*, paired recordings have identified ascending interneurons (aINs) as the source of glycinergic inhibition to dlc interneurons during swimming (Li et al., 2002). The zebrafish homologs of aINs, identified by the common expression of the transcription factor engrailed, are the circumferential ascending (CiA) interneurons (Higashijima et al., 2004b; Li et al., 2004). However, recordings from CiAs in the 20–29 hpf embryo do not show spontaneous action potential firing despite the high frequency of glycinergic activity seen in spinal neurons at this age (Saint-Amant and Drapeau, 2001; and our unpublished data). Furthermore, lesion experiments in the 24 hpf embryo have shown that glycinergic inputs to caudal CoPAs as well as to motoneurons are lost following a transection of the spinal cord at the eighth to tenth rostral spinal segment (Pietri et al., 2009), suggesting that rostral spinal cord or hindbrain neurons with descending axons, and not CiAs, are the source of glycine. By this same logic, it is unlikely that the glycinergic commissural local (CoLo) interneurons responsible for providing reflex inhibition to Mauthner cells during larval escape behaviors are the source of glycinergic inhibition to CoPAs since their axons span only one somite in length (Satou et al., 2009). A recent study by Moly et al. (2014) showed that a small number of glycine immunoreactive neurons are present in the hindbrain and rostral somites of the spinal cord at 20 hpf, coinciding with the arrival of glycinergic inputs to spinal neurons (Saint-Amant and Drapeau, 2000). One class of spinal neurons, the pacemaker IC cells, is located exclusively in the rostral spinal cord (Mendelson, 1986), suggesting that this region may contain specialized neurons not present in more caudal regions. Finding the corollary discharge neurons that provide glycinergic input to CoPAs and other neuronal classes will contribute greatly to our understanding of the developing spinal locomotor network.

### CoPAs AND OTHER NEURONS IN THE SENSORY PATHWAY ARE EXCLUDED FROM THE ELECTRICALLY COUPLED NETWORK

Our recordings from CoPAs in the 24–29 hpf embryo showed no evidence of electrical depolarizations, suggesting that these cells are not connected to the electrically coupled ipsilateral network driven by periodically depolarizing pacemaker neurons. In contrast, we have shown that the morphologically similar CoSAs show robust electrical depolarizations resembling those seen in motoneurons that can drive sustained action potential firing at this age. These findings appear inconsistent with a previous study in 20–24 hpf zebrafish embryos that reported the presence of electrical depolarizations in the majority of CoPAs but only a small minority of CoSAs (Saint-Amant and Drapeau, 2001). It is unlikely that these two cells types change their coupling to the network at 24 hpf, therefore we believe that CoPAs, having a very similar morphology at early ages to CoSAs, were misidentified. We are confident in our identification of CoPAs and CoSAs in this report because at these later stages, the lateral dendrites and large, triangular soma of CoPAs are obvious and easily distinguishable from the smaller soma of CoSAs that have no dendrites (**Figure 1B**).

It has been suggested that the dorsoventral localization of cell types may correlate with their inclusion in the electrically coupled network (Carlisle and Ribera, 2014). Unambiguously identifiable, dorsally located RBs and dorsal lateral ascending (DoLA) interneurons never show electrical depolarizations during the period of 20–29 hpf (Saint-Amant and Drapeau, 2001; Douglass et al., 2008; our unpublished observations), which is a predictable result for cells in the sensory pathway that should mediate evoked and not spontaneous, pacemaker-driven behaviors. We believe that CoPAs are not electrically coupled for these same reasons: their lack of observed spontaneous electrical depolarizations, their dorsal soma position, and their proposed role in sensory gating.

### STRONG AMPAergic EXCITATION ALLOWS BRIEF ACTIVATION OF CoPA INTERNEURONS IN RESPONSE TO EXTERNAL TOUCH STIMULI PRIOR TO THE ONSET OF SHUNTING GLYCINERGIC INHIBITION

Despite the evidence that CoPAs are not excited by the spontaneously active electrical network and in fact spend much of their time in a shunted mode due to the effects of their slow glycine receptors, CoPAs can nonetheless mediate a robust touch response prior to shunting *via* glutamatergic signaling. It has been established in *xenopus* that the glutamatergic currents from RBs onto dlc interneurons are mediated mostly by AMPARs, while the glutamatergic currents from dlc interneurons onto downstream motor and premotor interneurons are mediated mostly by NMDARs (Li et al., 2003). Furthermore, a single RB may synapse onto several if not all sensory interneurons of the ipsilateral spinal cord (Pietri et al., 2009; Roberts et al., 2010). The functional significance of these findings is that a few sensory neurons can strongly excite many sensory interneurons, but the relatively weak NMDAR-mediated responses in downstream neurons will not lead to activation of the network unless convergent inputs from sensory interneurons summate and reach threshold (Li et al., 2003).

Our results support these findings in several ways. First, we have shown that following a touch response, CoPAs receive strong, brief glutamatergic inputs with short latencies indicative of monosynaptic connections from RBs that lead to the robust firing of a single overshooting action potential. Secondly, RBs appear to be capable of producing only a single spike at this age (unpublished observations) therefore their synapses onto the CoPA must be strong to ensure that this single spike can activates the touch reflex pathway before shunting glycinergic inputs arrive. Finally, this activation appears to be mediated by AMPARs since we saw neither a reversal of glutamatergic currents at positive potentials nor any change in mEPSC kinetics in the presence of APV.

In neurons such as CoPAs which appear to lack a significant population of NMDARs, calcium-permeable AMPARs are likely to play a key role in raising cytosolic calcium levels needed for neuronal maturation and plasticity (Bowie, 2012). Calcium-permeable AMPARs are highly expressed at early developmental stages but are mostly replaced by AMPARs containing one or more GluR2 subunits later in development (Aizenman et al., 2002; Migues et al., 2007; Patten and Ali, 2007; Brill and Huguenard, 2008). Our results present new evidence showing that CoPAs express strongly inwardly rectifying AMPARs indicating calcium-permeability (Iino et al., 1990). Additionally, many of these AMPARs were insensitive to polyamine blockade by PhTX, suggesting that they may belong to a recently described, functionally distinct category of AMPARs that are both calcium-permeable and PhTX-resistant (Bowie, 2012). Previous studies have identified the presence of PhTX-insensitive, calcium-permeable AMPARs in the mammalian retina and hippocampus (Osswald et al., 2007; Mattison et al., 2014). In the developing retina the expression of these receptors coincided with the onset of activity in the visual system, suggesting that these novel AMPARs could play a role in synapse maturation. It will be interesting to determine whether the expression of PhTX-insensitive AMPARs in CoPAs coincides with the appearance of early spontaneous and touch-evoked behaviors and if expression is absent in animals in which sensory inputs have been blocked, as has been shown in the retina under conditions of dark-rearing (Osswald et al., 2007).

### SENSORY GATING IN OTHER SPINAL NETWORKS

Many predictions regarding the function of different neuronal classes in spinal locomotor systems are based on our knowledge from invertebrates (reviewed by Marder et al., 2005) as well as other simple vertebrates such as lamprey (reviewed by Grillner, 2003) and *xenopus* (reviewed by Buchanan, 2001 and Roberts et al., 2010). In *Caenorhabditis elegans*, an activated touch reflex circuit driving forward movement provides inhibition to command neurons in the reflex circuit that would otherwise drive backward movement and *vice versa* in order to prevent activation of both circuits at once (Chalfie et al., 1985). During touch-evoked swimming in the *xenopus* tadpole, the dlc sensory interneuron receives postsynaptic inhibition from ascending glycinergic interneurons that suppresses sensory transmission and prevents ongoing activation of the touch reflex (Sillar and Roberts, 1988). Our results reveal that the embryonic zebrafish spinal circuit uses a similar corollary discharge mechanism to inhibit touch reflexes during coiling and swimming. The synaptic properties of CoPAs are likewise tuned to neuronal function as demonstrated by the presence of fast AMPA receptors that drive strong activation following a touch stimulus and slow glycine receptors that mediate lost-lasting shunting. We find physiological evidence that CoPAs express rare glycine receptors with very slow kinetics and decreased strychnine sensitivity that may correspond to a unique subunit found in neonatal mammalian spinal cord. These findings suggest that both inhibitory corollary discharge and the specialization of postsynaptic receptors are common features of sensory gating in vertebrates. There remains a much greater complexity in dissecting these mechanisms in the mammalian spinal cord, but the recent description of transcription factors defining nine different types of mechanosensory interneurons in the rodent spinal cord (Del Barrio et al., 2013) may provide a tool with which to assess the functional roles and synaptic properties of common sensory interneurons classes across vertebrate species (Jessell, 2000; Goulding, 2009).

## AUTHOR CONTRIBUTIONS

Laura D. Knogler and Pierre Drapeau designed research; Laura D. Knogler performed research, analyzed data, and drafted manuscript; Laura D. Knogler and Pierre Drapeau edited and revised manuscript.

## Conflict of Interest Statement

The authors declare that the research was conducted in the absence of any commercial or financial relationships that could be construed as a potential conflict of interest.
